# Estrogenic regulation of hippocampal inhibitory system across lifespan

**DOI:** 10.1111/jne.13441

**Published:** 2024-08-15

**Authors:** Pablo Méndez, Rut de la Vega‐Ruiz, Alberto Montes‐Mellado

**Affiliations:** ^1^ Instituto Cajal (CSIC) Madrid Spain

**Keywords:** estrogen, hippocampus, inhibitory neuron

## Abstract

Estrogens produced in peripheral tissues and locally in the brain are potent neuromodulators. The function of the hippocampus, a brain region essential for episodic memory and spatial navigation, relies on the activity of ensembles of excitatory neurons whose activity is temporally and spatially coordinated by a wide diversity of inhibitory neurons (INs) types. Over the last years, we have accumulated evidence that indicates that estrogens regulate the function of hippocampal INs through different mechanisms, including transcriptional regulation and rapid nongenomic signaling. Here, we argue that the well‐documented influence of estrogens on episodic memory may be related to the actions of local and peripheral estrogens on the heterogenous populations of hippocampal INs. We discuss how physiological changes in peripheral sex hormone levels throughout lifespan may interact with local brain sources to regulate IN function at different stages of life, from early hippocampal development to the aging brain. We conclude that considering INs as mediators of sex hormone actions in the hippocampus across the healthy life span will benefit our understanding of sex‐biased neurodevelopmental disorders and physiological aging.

## 1 ESTROGENS AND THE HIPPOCAMPUS

Sex hormones represent one of the most powerful mechanisms by which biological sex impacts brain physiology.^1^ Estrogens, essential organizers of female reproductive function, deeply influence neuronal function by binding to different types of membrane and nuclear receptors expressed throughout the brain. Although classic studies mostly focused on addressing sex hormone effects in brain regions involved in reproductive behavior, we currently have solid evidence of the impact of estrogens on cognitive areas of the brain of male and female mammals.^2^, ^3^ The hippocampus, a cortical region involved in episodic memory and spatial navigation, has been shown to be a central node for the effects of estrogens in cognition. Several lines of evidence have demonstrated that estrogens have a profound impact on gene expression,^4^ synaptic plasticity^5^ and neurogenesis^6^, ^7^ of hippocampal excitatory neurons (ENs), the most abundant neuronal type in this brain structure. Importantly, normal hippocampal function relies on several relatively less numerous populations of inhibitory neurons (INs) that work in concert to support hippocampal function such as memory and spatial navigation.^8^ Through the release of the neurotransmitter gamma‐aminobutyric acid (GABA), these diverse groups of INs control the activity of adult ENs and play a critical role in the spatiotemporal organization of hippocampal activity. Although they are relatively less characterized in this regard, several lines of evidence point to a role of estrogens in regulating the function of INs. For example, the expression of estrogen receptors (ERs) has been described in both somatic and axonal compartments of hippocampal INs.^9^ Estrogens have a deep influence on several forms of hippocampal synaptic and network activity that critically depend on IN function.^10^ Moreover, estrogens influence several aspects of hippocampus‐dependent spatial, episodic, and emotional memories known to be dependent on the activity of INs.^3^ The essential role of INs in hippocampal function, as well as their importance in shaping normal development and aging effects in this brain region, makes this neuronal type a potentially important player in sex hormone regulation of cognitive brain function throughout the life‐span.

In this review, we aim to highlight the role of hippocampal INs as targets of estrogens and share our current view of IN's critical role in sex hormone regulation of hippocampal function. We discuss how the constantly changing sex hormone milieu may impact the heterogenous population of INs in different stages of life, from the organizational effects in early hippocampal development to activational effects in the adult and in the aging brain. Elucidating the interplay between peripheral and central sources of estrogens in the control of discrete neuronal populations will be useful to understand the sex‐specific effects of estrogens in brain function. Moreover, it will pave the way to improve sex‐hormone‐based therapies, to define pathological mechanisms of sex‐biased mental diseases, and to find strategies for buffering the combined effects of reproductive senescence and aging on brain function.

## 2 INs SUPPORT HIPPOCAMPAL FUNCTION

**FIGURE 1:**
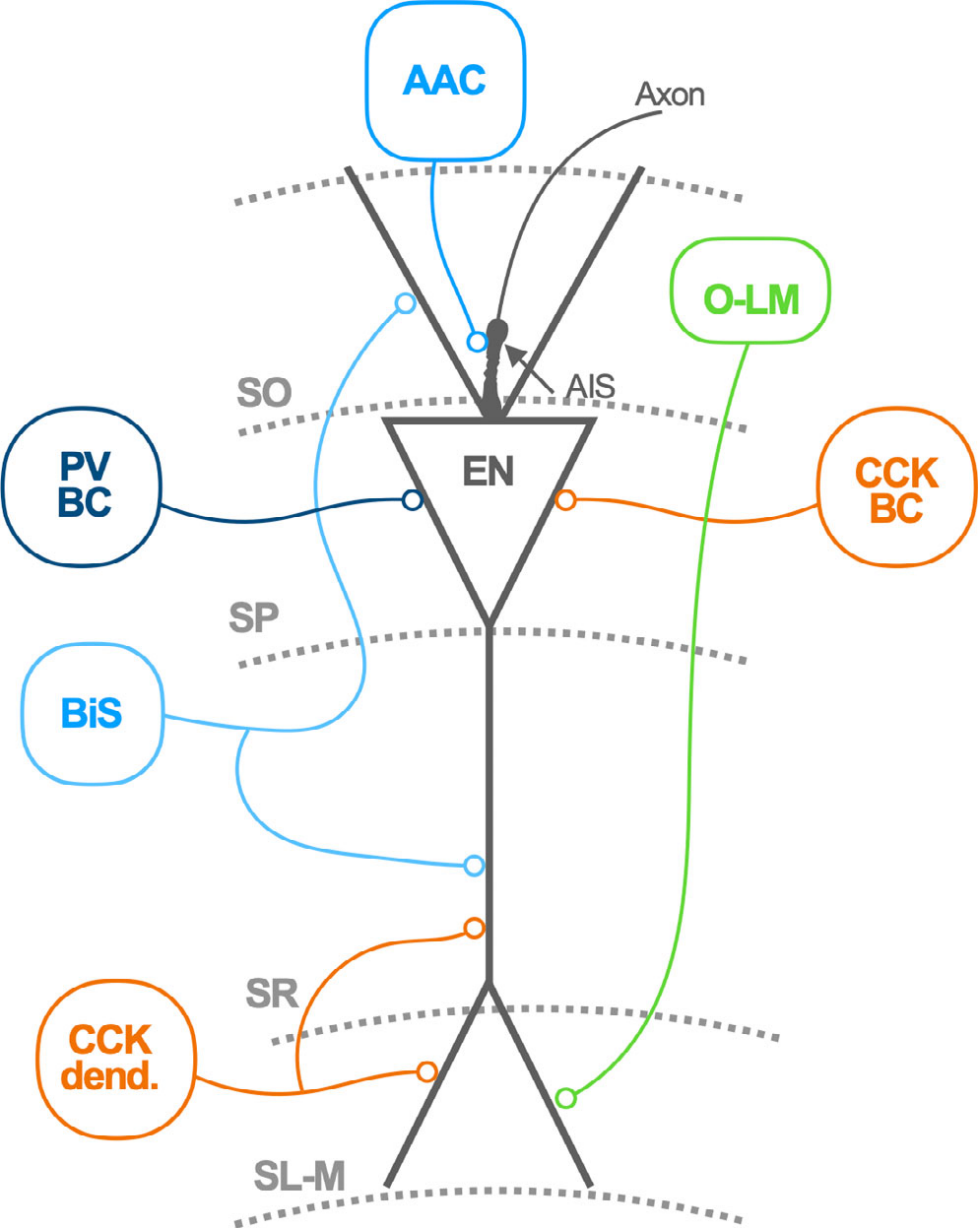
Hippocampal inhibitory neuron diversity. Simplified overview of hippocampal INs according to main morphological features, molecular marker expression, and target specificity in different compartments of postsynaptic excitatory neurons. For a detailed description of IN types in the study by Tzilivaki et al.^15^ EN, excitatory neuron; AAC, axoaxonic cell; O‐LM, Oriens‐Lacunosum Moleculare cell; PV BC, parvalbumin basket cell; CCK BC, cholecystokinin basket cell; BiS, Bi‐stratified cell; CCK dend., cholecystokinin dendrite‐targeting cell; SO, stratum oriens; SP, stratum pyramidale; SR, stratum radiatum; SLM, stratum lacunosum‐moleculare.

Hippocampal GABA‐releasing INs, which represent 10%–15% of total neurons, show a remarkable diversity that stems from early embryonic brain development.^11^ Morphological, electrophysiological, and molecular criteria^12^ and, more recently, transcriptomic techniques,^13^ allow for the distinction of dozens of different classes of INs. The function of the stunning diversity of hippocampal IN types is currently under intense investigation. IN subclasses are specialized in forming structured microcircuits with ENs and other INs (Figure 1). For example, two different subclasses of INs collectively known as basket cells, defined by the expression of the molecular marker parvalbumin (PV basket cells) or cholecystokinin (CCK basket cells) are specialized in forming synapses in the somatic compartment of hippocampal ENs.^14^ Other subclasses of INs expressing PV comprise (i) axoaxonic cells, which contact the axon initial segment, and (ii) bi‐stratified INs, which contact the dendrites of pyramidal cells in the oriens and radiatum strata. In contrast, the distal dendrites of pyramidal neurons in the stratum lacunosum‐moleculare are innervated by the axons of O‐LM cells, whose soma resides in the stratum oriens layer and express the markers somatostatin (SST) and, in some cases, PV. Distal dendrites of ENs are heavily innervated by yet another type of IN expressing the marker CCK.^15^

The relevance of this structured connectivity between the diverse INs and ENs becomes evident when assessing hippocampal activity during spatial navigation and memory processes. Several studies have demonstrated selective engagement of INs in different forms of physiological hippocampal activity, i.e., oscillations, underlying specific behavioral states.^12^, ^15^ Moreover, perturbing IN function with pharmacogenetic, optogenetic, or molecular tools has unveiled their role in specific aspects of hippocampal mnemonic function. CCK+ INs control memory precision during recall,^16^ SST INs determine memory stability by gating the activity of granule cells, a specialized type of EN in the dentate gyrus of the hippocampus.^17^, ^18^ PV and SST INs differentially regulate the activity of CA1 and DG ENs when mice explore new or familiar environments.^19^ In addition to episodic memory, the hippocampus has an essential role in spatial navigation that involves the activation of ENs in the CA1 region of the hippocampus at specific locations in the environment (place cells^20^). Place cell activity, which collectively forms a spatial cognitive map, is under the tight control of INs that influence the remapping of this activity when the environment changes.^21^, ^22^, ^23^

In summary, the diversity of INs is critical to organizing the wide variety of hippocampal activity modes observed in behaving animals and has a profound impact on the behavioral outcomes of these activities during spatial navigation and memory formation.

## 3 INHIBITORY NEURONS ARE SOURCES AND TARGETS OF ESTROGENS IN THE ADULT HIPPOCAMPUS

Estrogens acting in the hippocampus are produced in both peripheral tissues (mainly in male and female gonads) and in brain cells. Neurons from different brain regions, including ENs of the hippocampus, possess the enzymatic machinery required to transform cholesterol into sex steroids and produce the so‐called neurosteroids.^24^ Production of neuroestrogens by hippocampal ENs is well documented^25^, ^26^ but the contribution of INs remains to be fully described. The enzyme aromatase transforms testosterone into 17β‐estradiol, the rate‐limiting step in estrogen synthesis. Using immunohistochemical analysis of human, monkey, and rodent hippocampus, the presence of aromatase protein has been observed in hippocampal PV INs.^27^, ^28^, ^29^ In the mouse hippocampus, aromatase mRNA and the protein have been detected in INs expressing PV and SST.^30^ In the CA1 region, PV INs show higher expression levels compared with ENs and other IN classes such as SST INs.^30^ Using specific molecular markers to differentiate PV INs into different types, aromatase expression has been shown to take place preferentially in PV basket cells that show relative higher aromatase expression compared to other types of PV INs. The expression of aromatase and the accumulation of 17β‐estradiol^30^ suggest that hippocampal INs are a source of estrogens and may contribute, together with ENs, to local synthesis of neuroestrogens. Moreover, the different levels of aromatase expression by specific IN subclasses suggest differential contributions to hippocampal estradiol synthesis. Finally, the expression of aromatase in PV INs in human, monkey, and rodent brain points to a conserved functional role of neuroestrogen synthesis by this IN class.

Estrogens bind to different types of receptors, located in the membrane and cell nucleus, which trigger appropriate cellular and physiological responses. Nuclear receptors ERɑ and ERβ are transcription factors that regulate the expression of multiple genes over the course of several hours or days through the so‐called genomic effects. In contrast, the membrane receptor GPER (G protein‐coupled estrogen receptor) triggers rapid responses by activating intracellular signaling pathways (nongenomic effects). ERs are widely expressed among hippocampal ENs and glial cells.^2^ Importantly, the expression of ERs has also been reported in INs of the hippocampus. ERɑ expressing GABAergic INs were detected in different layers of the hippocampal subregion CA1, from Stratum Oriens to Lacunosum Moleculare.^9^, ^31^ More detailed analysis has shown that ER⍺ INs also express the marker CCK.^31^ ERɑ has also been detected in INs expressing the molecular markers Neuropeptide‐Y and PV.^10^, ^32^ On the other hand, studies in both rodents and monkeys have shown that ERβ is expressed in nonpyramidal neurons expressing the marker PV in the hippocampus.^33^, ^34^ To the best of our knowledge, GPER expression in hippocampal INs has not been reported to date. ERɑ and ERβ have also been detected at extranuclear locations, suggesting the existence of membrane‐bound forms of these receptors coupled to diverse cellular signaling pathways and rapid nongenomic mechanisms.^35^ Although this evidence provides a partial picture of ER expression in hippocampal INs, it suggests that estrogens may differentially impact different IN classes through the activation of different types of ERs. Further studies are required to define differential ER expression in molecularly identified INs in male and female hippocampus and to relate this expression to well‐known sex differences in the regulation of hippocampal function by estrogens.^36^

## 4 ESTROGENIC REGULATION OF INs: FROM GENE EXPRESSION TO PHYSIOLOGY

Similar to ENs, genomic and nongenomic actions of estrogens are involved in the regulation of hippocampal IN function. Several lines of evidence suggest that estrogens regulate the expression of glutamic acid decarboxylase (GAD),^31^, ^37^, ^38^, ^39^ the enzyme responsible for GABA synthesis, in adult rodent female hippocampus. In addition, the reduction of peripheral hormone levels through ovariectomy reduces PV expression in the dorsal hippocampus of female mice, an effect that is reversed by restoring the level of estrogens.^32^

Estrogenic regulation of gene expression in INs has been proposed to have functional consequences. The reduction in GAD levels observed after treatment with estrogens is paralleled by a reduction of functional inhibition, i.e. inhibitory synaptic activity, onto CA1 ENs.^31^, ^40^ The similarities in the time course and sensitivity to ER antagonism between estrogen‐induced reduction in GAD levels and the well‐known estrogenic effect in enhancing EN function have promoted the hypothesis that estrogens increase EN and glutamatergic synapse function through a genomic mechanism that involves transient reduction of inhibitory synaptic activity.^41^ The electrophysiological signature of estrogen‐mediated disinhibition of pyramidal neurons suggested changes in the presynaptic GABA release machinery, which is in line with ER expression in hippocampal INs and suggested direct actions of ER in this cell type, although the precise IN class and ER involved remain to be established.

The complexity of estrogenic actions in the hippocampus is illustrated by the fact that estrogen signaling in ENs indirectly affects IN function. Activation of ERɑ in the CA1 region of the hippocampus triggers a rapid effect that reduces the activity of a subset of GABAergic synapses impinging onto ENs.^42^ The rapid onset of this effect suggests a nongenomic mechanism of action. Indeed, pharmacological and biochemical experiments show that this effect is mediated by estrogen‐initiated signaling at metabotropic glutamate receptors (mGluRs) expressed by ENs. The coupling of membrane ERɑ and ERβ with mGluRs to trigger G protein signaling is an essential part of ER actions across the brain.^43^ In CA1 ENs, this mechanism triggers the activation of an inter‐neuronal retrograde signaling pathway mediated by the postsynaptic production of the endocannabinoid anandamide and its binding to cannabinoid receptor type 1.^42^ Interestingly, the expression for the CB1R defines a particular class of hippocampal INs that also express the neuropeptide CCK and the ɣ‐synuclein gene.^44^ This suggests that estrogens are selectively regulating a subset of CA1 PYR GABAergic synapses, those formed by CCK/ɣ‐synuclein/CB1R+ basket INs.

Most of the previously mentioned studies addressing the effects of estrogens on inhibition have used exogenously applied 17β‐estradiol. Although the concentrations used are compatible with those reported in brain tissue, whether local brain synthesis or peripheral sources of estrogens regulate synaptic inhibition remains to be investigated. A recent study describes how neuroestrogens, regulate the function of hippocampal PV INs and the activity of inhibitory synapses on CA1 ENs. The effects of neuroestrogens were studied in ovariectomized female mice, which lack the main source of peripheral estrogens, through the delivery of aromatase pharmacological inhibitors directly into the brain. The results show that neuroestrogens reduce synaptic inhibition in CA1 ENs through the regulation of perineuronal nets (PNNs), specialized extracellular structures that surround PV basket IN.^30^ PNNs are formed by a hyaluronan backbone to which different proteoglycans bind and regulate the synaptic and intrinsic excitability of PV INs.^45^ Enzymatic digestion of PNNs prevents the regulation of CA1 synaptic inhibition by neuroestrogens and disrupts the plasticity of hippocampal networks and hippocampal‐dependent cognitive behavior.^30^ Interestingly, PNNs across the brain show important sex differences and are regulated by the estrous cycle^46^ and stress,^47^ suggesting that they may represent a common mechanism for estrogenic regulation of neuronal function across different brain regions where these extracellular structures are present.

The evidence so far suggests that peripheral and brain‐derived estrogenic effects reduce the function of inhibitory synapses originating in two different types of INs: CCK/CB1 and PV‐expressing basket INs. The picture regarding the impact of estrogens in IN physiology is likely to be incomplete since other types of INs express aromatase and ERs. How different sources of estrogens interact in the control of INs remains to be established. It is possible that different IN classes may differentially sense local or peripheral sources to adapt hippocampal activity and function to specific conditions. Autocrine actions of estrogens in INs are suggested by aromatase expression in INs and would require co‐expression of ERs in the same estrogen‐producing cells. In principle, the lipophilic nature of 17β‐estradiol molecule poses certain constraints, due to limited solubility, to the passive transport across the extracellular space^48^ but, together with free diffusion through the plasma membrane and putative active transport,^49^ could support paracrine actions of estrogens across different neuronal and glial cell types in the hippocampus. In females, the estrous cycle imposes fluctuating levels of systemic estrogens with important consequences on the physiology and structure of excitatory hippocampal synapses.^50^ Moreover, the estrous cycle is also associated with changes in hippocampal estrogens levels^51^ and in the secretion of gonadotrophin‐releasing hormone, which in turn regulates the production of neuroestrogens in the hippocampus.^52^ Both phenomena may impact the function of specific IN subtypes. For example, recent studies have described increased density and intensity of PNNs surrounding CA1 PV INs in the proestrus stage of the estrous cycle, associated with high hormonal plasma levels.^47^, ^53^ The regulation of PV IN function and PNNs by neuroestrogens is independent of the endocrine function of the gonads since it takes place in ovariectomized female mice,^30^ but the influence of estrous cycle stage in the activity of PV INs or other hippocampal INs subtypes has not been directly addressed. Future studies have the potential to unveil a role for other fluctuating hormones, for example, progesterone, in regulating the function of defined subclasses of hippocampal INs.

Another important aspect that emerges from the study of estrogenic actions on INs is the sex‐specificity. The exogenous application of 17β‐estradiol reduces GABA release from presynaptic CCK INs only in brain slices obtained from female mice, but not from male mice due to sex differences in the molecular mechanisms engaged by 17β‐estradiol to regulate endocannabinoid synthesis and breakdown.^54^ Similarly, the blockade of neuroestrogen synthesis regulates PV INs PNNs and synaptic inhibition only in female mice.^30^ Although this seems to be a common principle for estrogenic regulation of hippocampal inhibition, the origin and the processes driving the sexual differentiation of the hippocampal inhibitory system regulation by sex hormones are not fully understood. The origin of this sex difference does not seem to involve differences in the local source of hormone, since aromatase expression in INs and ENs of the hippocampus is present in both sexes.^55^ However, aromatase activity is regulated by posttranslational mechanisms (i.e., phosphorylation) in an activity and estrogen‐dependent manner^56^ and some sex differences have been detected.^57^ Moreover, reducing peripheral sex hormone levels by castration in male mice uncovers a deleterious effect of the blockade of the synthesis of estrogens in hippocampal cognitive function.^58^ Similarly, aromatase blockers are effective in reducing pharmacologically induced epileptic activity in the hippocampus of male and female mice.^59^ Estrogens are well known to increase neuronal activity by facilitating excitatory synaptic transmission^3^ and thus, worsening hyper‐excitability in the epileptic brain. An intriguing possibility is that this protective effect of aromatase blockers in mouse models of epilepsy additionally arises from preventing the disinhibitory effects of neuroestrogens in INs. Altogether, this evidence suggests that different hormonal and activity‐dependent regulatory mechanisms influence estrogenic regulation of hippocampal INs in male and female mice.

## 5 ESTROGENIC REGULATION OF ADULT HIPPOCAMPAL NETWORK ACTIVITY AND FUNCTION THROUGH INs

The diverse types of IN organize the activity of hippocampal neural networks and provide an extremely precise temporal code used by neurons to process and store information.^15^ Different types of hippocampal oscillatory activities predominate under specific behavioral states and are critically related to higher order cognitive functions. For example, Ɣ and ϴ oscillations emerge during active exploration, and locomotion and are important for information encoding and binding in specific neuronal ensembles.^20^, ^60^ High‐frequency sharp‐wave ripples are frequent (SWR) during immobility and sleep have been linked to the process of memory consolidation in neocortical regions.^61^

**FIGURE 2:**
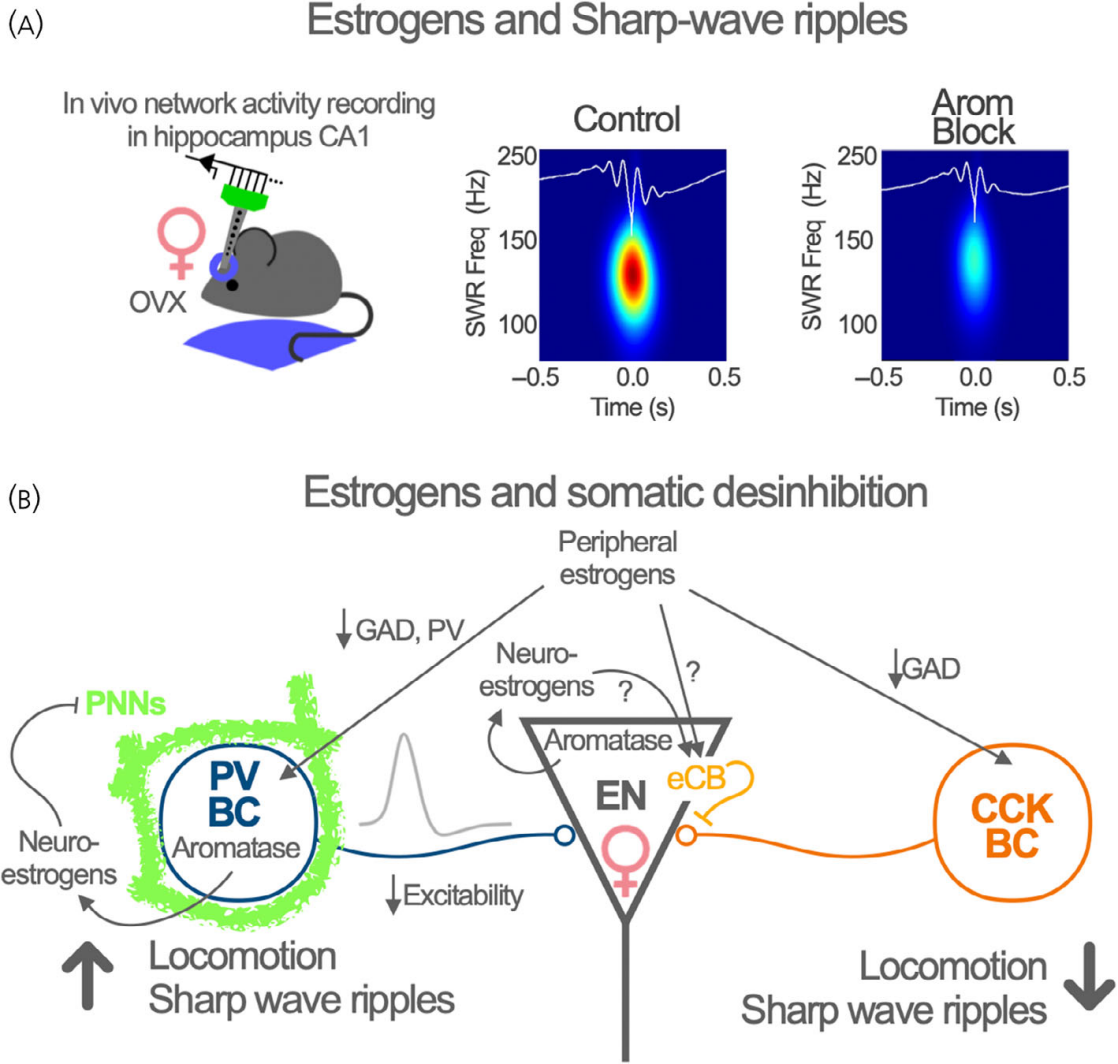
Estrogens and functional regulation of hippocampal inhibitory neurons. (A) Estrogens impact on hippocampal inhibition is suggested by the effects of neuroestrogens synthesis blockade by pharmacological inhibition of aromatase activity (Arom Block) on Sharp Wave Ripples recorded in awake ovariectomized (OVX) female mice. Adapted with permission from the study by Hernández‐Vivanco et al.^30^ (B) Somatic targeting basket cells (BC) expressing the molecular markers parvalbumin (PV) and cholecystokinin (CCK) are regulated by estrogens. The figure shows estrogens regulated actions on the gene expression (GAD, Glutamic Acid Decarboxylase; PV, parvalbumin), perineuronal nets (PNNs), inhibitory synapses, and intrinsic excitability. eCB, endocannabinoid. The association of PV and CCK BCs with behavioral states (locomotion) and network oscillations (Sharp Wave Ripples) is represented in the lower part of the panel. See details in the main text.

The evidence regarding the effects of estrogens on hippocampal oscillations is scarce. A pioneer study evidenced that ovariectomy produces a significant reduction in hippocampal Ɣ oscillations during decision‐making in a Y maze, a behavioral test relevant to spatial memory.^10^ Interestingly, peripherally administered estrogens and raloxifene, a selective estrogen receptor modulator frequently used in clinics, enhance oscillations and recover performance of OVX female mice, suggesting a relationship between gonadal estrogenic modulation of hippocampal network oscillations and the performance in behavioral tests.^10^ A more recent study assessed the effects of neuroestrogens in different types of hippocampal oscillations recorded in awake, head‐restrained female mice.^30^ Pharmacological suppression of estrogens synthesis by aromatase was paralleled by a reduction in the power of ϴ, Ɣ oscillations as well as in SWRs (Figure 2A). Since these effects were observed in ovariectomized female mice, the results point to a role of neuroestrogens in regulating these forms of activity, likely through the functional impact on PV INs. As expected from the critical role of these oscillations in information processing, storage, and recall, these effects were accompanied by a sex‐specific reduction in the performance of female mice in a hippocampal‐dependent spatial memory task.^30^

As stated above, estrogens reduce the activity of different subtypes of basket cells INs (PV and CCK). Interestingly, although these two complementary IN subtypes target the same subcellular compartment of ENs, the perisomatic compartment, the input and output connectivity, and their regulation by neuromodulatory systems such as cannabinoids and opioids,^14^, ^62^ are largely different. Moreover, the activity of PV and CCK basket cells is differently affected by behavior. While CCK IN activity tends to increase when the mice reduce locomotion and eventually stop moving, the activity of a large majority of PV INs increases when mice start moving.^44^, ^63^ By acting on these cell types, peripheral and local estrogens may disinhibit the somatic compartment of ENs across different behavioral states (Figure 2B). Neuroestrogens regulate PV INs activity during locomotion in female mice, suggesting that estrogens modulate the coupling of IN activity to behavior in vivo.^30^ Peripheral^64^ and local^65^ estrogens have direct effects on glutamatergic synapses of ENs but the sources regulating inhibitory synapses from CCK INs have not been identified. Moreover, whether estrogens regulate the engagement of PV and CCK IN activity during the specific forms of network activity that underlie these behavioral states remains to be determined. PV basket cells are active during SWR,^44^ raising the possibility that estrogenic signaling may regulate the proper balance between excitatory and inhibitory neurotransmission during these events. Thus, different sources of estrogens impacting CCK or PV INs may support or modulate structured microcircuits formed by hippocampal ENs, balancing information processing toward different task variables and controlling the balance between complementary memory representations.^66^

Estrogens have a profound impact on learning and memory processes that critically depend on hippocampal INs. In rodents, estrogen effects in spatial, episodic, and emotional memories have been tested in multiple hippocampal‐dependent tasks (Morris Water Maze, Barnes Maze, novel object location test, and Contextual Fear Conditioning). A frequently utilized strategy is surgical removal of the gonads (orchidectomy and ovariectomy) in conjunction with exogenous hormone replacement. In this way, gonadal‐derived estrogens have been shown to support episodic spatial memory in female and male rodents through multiple molecular mechanisms.^3^, ^67^ Importantly, infusion of aromatase blockers into the hippocampus of gonadectomized male or female mice impaired consolidation of spatial memory, suggesting a role for neuroestrogens in hippocampal‐dependent cognition.^30^, ^68^ In contrast to the wealth of evidence supporting the effects of estrogens in hippocampal function, we have only indirect evidence of the role of INs in mediating these actions. This is in part due to the scarcity of cell type‐specific approaches to monitor or intervene the synthesis and sensing of estrogens in INs during hippocampal‐dependent behavior. Conditional knock‐down strategies have been used to eliminate aromatase expression from forebrain ENs, including ENs in the hippocampus. Abolishing the synthesis of estrogens in forebrain ENs affected hippocampal‐dependent spatial and emotional memories and molecular and structural correlates of excitatory synapse plasticity related to learning and memory in both male and female mice.^65^ The important role of INs in learning, memory, and spatial navigation suggests that using similar conditional approaches targeting the synthesis and sensing of estrogens in INs would expand our knowledge about the neuromodulatory role of estrogens in these processes. Moreover, the reported sex differences in estrogenic regulation of INs discussed above suggest a potential for these approaches to explain sex effects in the strategy used to solve spatial tasks^36^ and also to increase our understanding of the influence of noncognitive aspects, such as stress^47^ in spatial and emotional memories.

## 6 IN AS TARGETS OF SEX HORMONES DURING DEVELOPMENT AND JUVENILE HIPPOCAMPUS

The organizational actions of sex steroids and their receptors around birth are responsible for the establishment of most sex differences in the brain.^69^ In the embryonic hippocampus and cortex, a complex set of genetic programs determine the specification and migration of INs.^11^ In addition, INs have a protracted postnatal maturation period that guides the wiring and integration in neuronal circuits.^11^ The temporal overlap with the critical period for sexual differentiation of the brain suggests a potential role of IN development in the sexual differentiation of the hippocampus.

The levels of estrogens^70^ and ERs expression^71^ are elevated in the postnatal hippocampus. Aromatase activity peaks after birth and remains elevated until the third week of age in the hippocampus of male and female rodents.^72^ Sex differences in reproductive behavior have been shown to depend on the metabolism of circulating, testis‐derived testosterone into estrogens by central aromatase.^73^ A plausible hypothesis is that a similar mechanism takes place in cognitive areas of the brain. Indeed, estrogens have been shown to promote survival, axonal growth, dendritic branching, and synaptogenesis of developing hippocampal neurons.^74^ Importantly, in some instances, estrogenic regulation of hippocampal neuron development has been shown to involve neurons releasing the neurotransmitter GABA.^75^ For example, ER activation prolongs the depolarizing effects of GABA in cultured rat hippocampal neurons,^76^ suggesting that estrogens may be responsible for the switch between early excitatory actions of GABA and the later inhibitory responses, impacting in this way network activity that shapes hippocampal postnatal maturation. The pioneering work of Murphy et al. described the downregulation of the GABA‐producing enzyme GAD in cultured embryonic hippocampal neurons through the activation of ER expressed in INs.^37^ This effect correlated with a decrease in BDNF expression,^41^ an important regulator of IN development.^77^ The regulation of BDNF levels by estrogens in the developing hippocampus in vivo^78^ could control the timing of maturation and functional integration of INs in hippocampal networks. In addition, estrogens and aromatase regulate the expression of reelin in organotypic slice cultures of the hippocampus.^79^ Reelin is a secreted protein produced by Cajal–Retzius cells that guide brain development by controlling cell‐to‐cell interactions.^80^ Although most Cajal–Retzius cells are eliminated during development, some of them persist in the adult brain where they are important organizers of hippocampal inhibition,^81^ representing an additional mechanism by which estrogens could be controlling the development of hippocampal INs.

Recently, aromatase protein expression has been described in hippocampal INs in young male and female mice at postnatal day 21, suggesting local production of estrogens. At this age and until puberty, the production of sex hormones by the gonads remains at very low levels. In contrast with this fact, pharmacological inhibition of aromatase has sex‐specific effects in synaptic inhibition onto CA1 pyramidal neurons in prepubertal mice, suggesting that neuroestrogens regulate IN function during the postnatal period.^53^ Akin to adult mice, the effect of aromatase inhibition was only observed in young female mice, not in male mice. In the postnatal hippocampus, INs control the refinement of network activity and the critical periods for plasticity.^11^, ^82^ Thus, neuroestrogens regulation of functional inhibition may represent a mechanism to shape the maturation of network activity in the female hippocampus. Moreover, testosterone exposure in female pups disrupts neuroestrogens regulation of synaptic inhibition and PV INs PNNs in the adult brain.^53^ Although it is currently unknown whether this effect is mediated by aromatization into 17β‐estradiol, long‐term effect of neonatal testosterone in the hippocampal inhibitory system resembles organizational effects in reproductive areas of the brain.

Later in life, puberty is associated with changes in hippocampal function and inhibitory synapses, reversing plasticity rules in a sex‐specific manner.^83^ Although the cellular mechanisms of this pubertal effect remain to be established, sex hormones are likely candidates due to their potential to regulate inhibitory synaptogenesis in the hippocampus and synaptic inhibition in neocortical neurons.^84^

## 7 IMPACT OF ESTROGENS IN IN DURING PHYSIOLOGICAL AGING

Aging is associated with cognitive decline in humans and rodents. In women, aging interacts with the loss of ovarian follicular function and the decrease of ovarian synthesis of estrogens during menopause, worsening cognitive decline and the incidence of neurodegenerative diseases.^85^ A significant part of menopause symptoms affects brain processes controlled by INs such as sleep, anxiety, and mood.^86^ In addition, cognitive symptoms are common among women transitioning through menopause. The incidence of memory difficulties during natural and surgically induced menopause is reported by up to two‐thirds of women.^87^ Eventually, interactions between age and menopause lead to neurodegenerative diseases such as Alzheimer's disease, which is more frequent and severe in women than in men.^86^ Decades ago, hormone replacement therapies (HRTs), which exogenously compensate for reduced ovarian sex hormone production, were proposed to counteract the consequences of natural and surgically induced menopause. Large clinical studies have shown that while HRTs show clear benefits for hot flushes and osteoporosis, they show mixed effects on improving brain function.^88^ Although it is now clear that specific HRT formulations and the onset of treatment play a significant role in the cognitive outcome, HRTs are not currently advised for cognitive symptoms of menopause. The conflicting results obtained from large clinical trials highlight our lack of knowledge about the basic mechanisms of action of sex hormones during physiological aging.

INs are deeply affected by aging. Expression of GAD is decreased in the aged hippocampus.^89^, ^90^ There is reduced expression of several IN markers^91^ and this reduction correlated with aged‐associated impairment in a hippocampal‐dependent spatial memory task.^92^ Moreover, aging is associated with a reduction in functional synaptic inhibition onto hippocampal ENs.^93^ Accompanying these alterations in the inhibitory system, and probably related to them, physiological aging affects hippocampal oscillations. Several studies have compared hippocampal gamma and theta oscillations in young and aged animals. Although these studies report no overt differences in the general properties of oscillations (for example the power), they showed that the coupling of these oscillations to animal locomotion was different in both age groups.^94^ Moreover, the high‐frequency oscillations SWRs are reduced with aging.^95^, ^96^ The critical role of INs in organizing hippocampal oscillations suggests that aging may be affecting IN function. Unfortunately, most of the studies assessing age effects on hippocampal oscillations have not systematically compared males and females. Arguably, the influence of reproductive senescence on the hippocampal network and its potential interaction with aging remains to be studied. In monkeys, a suitable model for studying reproductive senescence, the levels of hippocampal ERβ mostly expressed by PV+ INs, are increased after menopause, suggesting enhanced sensitivity to estrogens.^34^ The expression of aromatase was unchanged after menopause, raising the possibility that neuroestrogens may be relevant in the control of IN function during physiological aging in female monkeys. How peripheral and brain estrogens interact with aging in supporting female hippocampal INs is currently unknown. Investigating the function of neuroestrogens in the aging hippocampus could allow us to propose strategies to mitigate the impact of aging and menopause on hippocampal‐dependent memory.

## 8 CONCLUSIONS AND FUTURE DIRECTIONS

It is increasingly clear that sex hormone‐related processes are integral parts of the basic mechanisms supporting learning and memory and a good example is the well‐known effects of estrogens in sustaining appropriate hippocampal physiology and plasticity underlying learning and memory. The critical role of INs in supporting hippocampal cognitive function and the dynamic regulation of brain function by sex hormones throughout the life span highlights the potential relevance of future studies aimed to define how estrogens impact IN function. In this review, we stressed the necessity of considering the cellular heterogeneity of hippocampal INs in order to understand the impact and interaction of local and peripheral estrogens in this brain region. We believe that neuron‐type specific strategies that exploit the wealth of genetic tools to target specific neuronal classes will be an invaluable resource to identify IN subtypes that act as sources of targets of estrogens. For example, several transgenic mice lines and viral vectors could be used to conditionally reduce the expression of aromatase and ERs in defined types of INs. Moreover, these same tools, in combination with surgical and pharmacological modulation of estrogen sources, could be used to monitor the activity of genetically‐defined INs during behavior to unravel the interaction of peripheral and local brain sources in the regulation of brain function. These approaches will allow us to advance our knowledge about potentially relevant questions:

- How do autonomous (autocrine) or paracrine actions of estrogens change the activity of the structured microcircuits formed by ENs and INs in the hippocampus?
- How does biological sex determine the actions of estrogens in different types of hippocampal neurons?
- What regulates aromatase activity during development and what are the long‐term consequences of aromatase activity in hippocampal INs during the postnatal period in males and females?
- How do aging and reproductive senescence affect the estrogenic regulation of INs?

Answering these questions would have a relevant impact on understanding how sex‐specific actions of estrogens support normal hippocampal neuronal network development and prevent alterations with long‐lasting consequences, such as those responsible for neurodevelopment disorders. Moreover, it could pave the way to leverage the potential of brain local synthesis of estrogens to mitigate cognitive symptoms of aging. Finally, estrogen‐based therapies are frequently used in clinics to treat a wide range of conditions. Understanding the mechanisms of action in the hippocampus may represent a useful step to minimize the negative effects of some sex hormone‐based therapeutics on cognitive function.^97^, ^98^

## AUTHOR CONTRIBUTIONS

**Pablo Méndez:** Conceptualization; funding acquisition; writing – original draft; writing – review and editing. **Rut de la Vega‐Ruiz:** Writing – original draft; writing – review and editing. **Alberto Montes‐Mellado:** Writing – original draft; writing – review and editing.

## FUNDING INFORMATION

This work was supported by a grant PID112428GB‐100 by MCIN/AEI/10.13039/501100011033 to Pablo Méndez. Alberto Montes‐Mellado is supported by a JAEIntro scholarship funded by CSIC. Rut de la Vega‐Ruiz is supported by the PhD fellowship PRE2021‐099806 funded by MCIN/AEI/10.13039/501100011033 by “ESF Investing in your future.”

## CONFLICT OF INTEREST STATEMENT

The authors declare no conflicts of interest.

## PEER REVIEW

The peer review history for this article is available at https://publons.com/publon/10.1111/jne.13441.

## Data Availability

Data sharing is not applicable to this article as no new data were created or analyzed in this study.
